# Towards culturally inclusive healthcare in Peru: Mapping epistemic concepts in contemporary Indigenous Amazonian medicine—Traditional healers’ perspectives

**DOI:** 10.1371/journal.pgph.0003912

**Published:** 2025-01-17

**Authors:** Ilana Berlowitz, Maria Amalia Pesantes, Cynthia Cárdenas Palacios, Chantal Martin-Soelch, Ursula Wolf, Caroline Maake

**Affiliations:** 1 Institute of Anatomy, Faculty of Medicine, University of Zurich, Zurich, Switzerland; 2 Unit for Clinical and Health Psychology, Department of Psychology, University of Fribourg, Fribourg, Switzerland; 3 Centre of Excellence in Chronic Diseases, Universidad Peruana Cayetano Heredia, Lima, Peru; 4 Institute of Human Sciences and Philosophy, Universidade Federal do Amazonas, Manaus, Brazil; 5 Institute of Complementary and Integrative Medicine, University of Bern, Bern, Switzerland; SRMIST: SRM Institute of Science and Technology (Deemed to be University), INDIA

## Abstract

Peru is among Latin American countries with the largest Indigenous population, yet ethnical health disparities persist, particularly in the Amazon region which comprises 60% of the national territory. Healthcare models that include Indigenous medicine and traditional healers present an important avenue for addressing such inequalities, as they increase cultural adequacy of services, healthcare access, and acknowledge Indigenous Rights for their perspectives to be represented in public healthcare. Understanding the underlying epistemologies of Indigenous medicine is a prerequisite for this purpose. Thus, in order to support Indigenous Organizations and governmental initiatives to develop more inclusive healthcare approaches, the current study investigated key epistemic concepts in Indigenous-Amazonian medicine from the perspective of traditional healers. We conducted systematic in-depth interviews (semi-structured) with a sample of 13 healers of three Peruvian-Amazonian regions (Loreto, Ucayali, San Martín). Data was analysed using manifest qualitative content analysis. Our findings point to an intricate medical system based on a sophisticated understanding of health, illness, and treatment. Indigenous healers described multifactorial aetiology concepts, complex interactions between material and spirit-related aspects of body and nature, diagnosis, and treatment. These often involved carefully designed applications of ‘teacher plants’, a concept at the heart of this medical system. Furthermore, while the healers considered traditional and biomedicine as complementary systems, they identified the lack of recognition of traditional healers as a primary barrier for collaboration. Indeed, preconceptions and stigma on Indigenous medicine along with a paucity of research, still represent an impediment to countries’ ability to respond to Indigenous peoples’ health-related expectations and needs, thus maintaining existing inequalities. This work offers a significant contribution to the understanding of Indigenous-Amazonian medicine and perspectives of traditional healers, relevant for Peru and adjacent countries sharing Amazonian territory and cultures. Our findings also highlight Amazonian healers’ unique expertise around the therapeutic applications of psychoactives, from which the current revival of clinical scientific interest in psychedelic-assisted therapies may have a great deal to learn.

## Introduction

Medical pluralism—the coexistence and parallel use of biomedicine and other medical traditions—is the global rule rather than the local exception [1,2]. Biomedicine services are functionally weak in most developing countries, where public health services especially in rural regions are often lacking, and those that do exist often fail to account for the local cultures’ characteristics [3–7]. In Latin America approximately a tenth of the population is Indigenous (from 62% in Boliva to 1% in Brazil [8]), representing around 42 million people; yet, in most countries they remain marginalized, live almost uniformly below the poverty line, and exhibit higher rates of morbidity and mortality than their non-Indigenous counterparts [9]. Persistent health disparities are among key expressions of social inequities in the region, suggesting an important need for increased investment in accessible, culturally adequate health services [10–17]. Indeed, in its mission to improve global health, the World Health Organization (WHO) has been promoting the recognition and inclusion of Indigenous or traditional medical systems as a necessity towards meeting the objective of Universal Health Coverage (UHC) [18–21]. Incorporating traditional medicines into the International Classification of Diseases-11 [22] has been part of these efforts, and the policy overall has pushed many countries to reflect upon Indigenous medicine perspectives and cultural pertinence of services, which is also a right of Indigenous Peoples as per United Nations Declaration on the Rights of Indigenous Peoples (UNDRIP) [23].

From an epistemological perspective, both biomedicine and Indigenous medicine are networks of linked actors producing medical knowledge, which is transformed into medical interventions at the individual/familial, community, or public level. While each is a knowledge system in its own right, they embody distinctive worldviews and perspectives. Biomedicine is the body of knowledge and practices developed in the West to face disease; it is the dominant medical system, but just as any medical system, the product of particular historical processes and cultural values [24]. Biomedicine builds on the body of Western scientific thought and technology, as well as highly elaborate organizational and educational resources. Treatments tend to focus on monocausal pathways, with attention to systemic pathways being fairly recent (e.g., psychosomatic interactions in chronic syndromes, biopsychosocial frameworks in mental health) [25–27]. In contrast, Indigenous or traditional medicines (used interchangeably in this paper), which the WHO defines as “knowledge, skill and practices based on the theories, beliefs and experiences indigenous to different cultures, whether explicable or not, used in the maintenance of health, as well as in the prevention, diagnosis, improvement or treatment of physical and mental illnesses” [p.8; 21], are generally in possession of less resources, less status, little institutional support, and are usually oral traditions. They tend to include extensive knowledge on herbal products, a focus on prevention and nutrition, and an integral approach towards body, mind, and spirituality [28]. They are often strongly embedded in community and cultural context, and tend to address both individual and collective well-being.

Peru is among the three Latin American countries with the highest percentage of Indigenous peoples, both in terms of absolute numbers and relative proportion [8,13]. Yet, discrimination and inequalities in healthcare are notorious, with Indigenous populations often lacking access to affordable and culturally appropriate services [29–33]. This is particularly evident in the Amazon region [34–36] which makes up 60% of Peru [37] and, spanning across nine countries, about 40% of South America. During the Covid-19 pandemic exceptionally high death rates in both the Peruvian [38–41] and the Brazilian Amazon [42–44] made the international headlines, pointing to great regional vulnerability (cf. syndemics [45]) and pressing need for improved healthcare. During this period, several grassroots initiatives of Indigenous volunteers (e.g., Shipibo, Ashaninka/Asheninka communities) emerged, attempting to respond to local health needs with Amazonian medicinal plants and approaches [39,46–48]. Indeed whereas the informal contribution of traditional healers may be vital [cf. 49], their formal integration in the public health sector has not yet been accomplished [50]. This would however be essential to improve acceptability of services and UHC [50], and for acknowledging Indigenous people’s rights to co-determine healthcare as per UNDRIP, of which Peru is a signatory [21,23,51–56]. Systematic research on underlying medical epistemologies of traditional Amazonian healing is an important prerequisite in this context, as it provides the basis for dialogue between medical systems and for the development of integrative care models and joint care routes. The present work thus aimed to describe key epistemic concepts [57] in Peruvian Amazonian traditional medicine from the perspective of local traditional healers.

In addition to Amazonian medicine’s significance for inclusive healthcare in South America, this traditional healing system is currently also globally discussed in conjunction with the ‘psychedelic renaissance’, the revival of clinical interest in psychedelics/psychoactives, described as the promising ‘next generation of psychiatric treatments’ [58–62]. Amazonian medicine is prominently referenced in this context mainly due to ayahuasca, a psychotropic herbal remedy (*Banisteriopsis caapi* and admixture plants) developed by Amazonian Indigenous healers for various indications, although, as evident in ethnobotanical studies, other local psychoactives are commonly employed in Amazonian medicine (e.g., *Brugmansia spp*. like toé) [63–68]. The popularity of ayahuasca has led to its global investigation in clinical studies (depression, anxiety, addictive and other disorders), with first RCTs pointing to beneficial therapeutic effects [69–74]. Yet, in spite of Amazonian traditional healers’ long-standing experience and intellectual ownership in this context, they are rarely participant in clinical research and associated developments [75–78], again symptomatic of the notorious structural exclusion of Indigenous actors and knowledge systems [79,80].

Thus, aiming to further the development of more inclusive approaches, this study investigated key epistemic concepts of Indigenous-Amazonian medicine from the perspective of traditional healers as experts. More specifically, based on in-depth interviews with experienced traditional healers from three regions of the Peruvian Amazon, we aimed to systematically describe emic perspectives on illness and health, etiology, diagnosis, and treatment.

## Methods

### Setting

This study was part of an international transdisciplinary research project (*InterAct-Health*; Swiss Programme for Research on Global Issues for Development) investigating new avenues for culturally pertinent public health provision systems in countries with large Indigenous populations, focusing on Peru and Guatemala (2018–2022). The study was conducted in accordance with international/national regulations and approved by the Institutional Review Board of the University of Fribourg, Switzerland (approval number: 88A2).

### Sampling

We used a consecutive sampling approach for participant recruitment, contacting potential informants via telephone, email, or face-to-face. Inclusion criteria were defined as being a traditional healer, having been born in the Peruvian Amazon, and having practiced their trade for at least ten years. Exclusion criteria comprised being a minor (age below 18). The ensuing sample consisted of 13 key informants who were experienced traditional healers (subsequently also ‘healers’, or Spanish: ‘*curanderos/as’*). All participants were thoroughly informed about the study and provided formal written and verbal consent.

### Data collection

During the recruitment period (1.10.2019–31.10.2021), in-depth interviews were conducted in Loreto, Ucayali, and San Martín, the three most populated departments of the Peruvian Amazon [81] by a health professional with extensive fieldwork experience in this region (IB). Interviews took place at the informants’ workplace or home and were held in Spanish, in which all participants were fluent (Spanish being the shared spoken language in the region, see history of colonial missionaries and extractive industries [82,83]). Given the time it took to cover the rather extensive list of topics (see next sections), the interviews in all but one cases were held in several sittings (2–3 sittings per respondent in most cases; a total of 37 sittings across the 13 participants), with individual variations to accommodate their specific time constraints, verbal styles, and preferences (e.g., some tended to speak more than others, some favoured multiple short sittings over few lengthy ones). Data collection was discontinued once the material derived from this procedure was considered suitably rich in terms of information power [84] (see also data adequacy [85]). For further details see the supplementary checklist with consolidated criteria for reporting qualitative studies [86].

### Instrument

To guide the data collection, we developed a semi-structured interview schedule using a procedure described by Kallio et al. [87]. The first author compiled an extensive list of sample questions covering relevant content areas based on the literature regarding traditional Amazonian healing, including our own, as well as the literature on medical epistemologies and related classification schemes [e.g., 57,88,89–107]. The item list was reviewed by the transdisciplinary advisory panel (scientific and practice experts from biomedicine, clinical psychology, traditional medicine—including traditional healers–, complementary medicine, and anthropology) and modified where necessary. The list format was then adapted to a semi-structured open-question format, which has the advantage of permitting the exploration of emergent topics and follow-up questions by both parties, thus increasing breadth and depths of elicited data [108]. The resulting interview guide was again reviewed by the expert panel and then pilot tested. The final guide consisted of main and sub-chapters, which in turn were made of questions and probing cues (main chapters: *demographic and professional information, current healing practice, the human make-up, concepts of illnesses* [note: the terms ‘illness’ and ‘disease’ are used interchangeably in the present scope]*, diagnosis, causes of illness, treatment, effectiveness*).

### Data analysis

The audio-recorded interviews (38.9 h in total; 180 min per interviewee on average) were transcribed verbatim and the textual material then analysed using a manifest content analytic approach [109–111] facilitated by qualitative analysis software (MAXQDA 2020 [112]). A preliminary set of themes deductively defined based on the interview guide was used to work through the material initially; meaning clusters that emerged in this process were inductively defined as subthemes. Over 1500 text segments were thus coded along the emerging category system using an iterative process. Where necessary, main themes were adapted (narrowed/broadened, rephrased, reallocated, split/merged), thereby gradually refining the coding system to maximize its capacity to represent the range of the data at hand. The resulting clusters of epistemic concepts will next be presented in condensed form, whenever possible using direct quotes to minimize deviations from the original expression of the healers (designated as *HA* [*Healer A*], *HB* [*Healer B*], etc.). While the analysis was performed in the language of the original material (i.e., Spanish), the selected quotes were then translated into English and in some instances slightly shortened to ensure informant anonymity and to keep space requirements minimal. Evidently, it is impossible to represent the breadth, depth, and nuance of an entire millennia-old medicine system in the scope of an academic article; the current work should thus be understood as an initial map of concepts that also sets foundations for further work.

## Results

### Key informants

All informants were born in the Peruvian Amazon (Loreto: 5, Ucayali: 5, San Martín: 3), identifying themselves as associated with one or several Indigenous-Amazonian traditions via their ancestry and/or medicine teachers (*Maestros*), including Awajun, Capanahua, Kichwa, Kukama, Kukama-Kukamiria, Murato, Shipibo, Shipibo-Conibo, and Yine (terms used by the informant). While some also spoke an Indigenous-Amazonian language, all informants were fluent in Spanish (first language for 85%). Most of them started learning Amazonian medicine during their childhood years (*M* = 14 years of age, range: 7–32; see Table 1), often under the tutelage of a close relative (parent, grandparent, uncle; e.g., “since I was little, 6, 7 years old, my mother who was a healer would tell me ‘go get this plant’, and we would go; sometimes we would cook the remedies, she would say ‘boil this’, or ‘prepare that as an infusion’, and that is how we began to learn the natural medicines’ *HA*). Their ages at the time of the interview ranged between 36 and 73 years (*M* = 55), except for one they were male, and reported an average of 28 years of clinical experience at the time of the study (range: 12–50). The informants started to work as healers at age 27 on average (range: 13–58), but emphasized that their training has never really ended (“the process of learning does not happen from one day to another, nor is it done within one or two or three months; it means dedicating one’s life to it” *HE*). Various areas of specialization within Amazonian medicine were reported, defined by their main plant or field of application (see also *2.2.* for further explanations). They included specialists in herbal remedies (*vegetalista*), tobacco-based treatments (*tabaquero/a*), ayahuasca-based treatments (*ayahuasquero/a*), tree-based remedies (*palero/a*), steam baths (*vaporador/a*), aromatic plants/substances (*perfumero/a*) or *camlonguero/a*, bone-setting (*sobador/a* or *huesero/a*), and midwifery (*partero/a*).

**Table 1:** Years of clinical experience and age of participants at relevant time points.

	HA	HB	HC	HD	HE	HF	HG	HH	HI	HJ	HK	HL	HM
Age at time of interview	73	46	54	72	36	50	53	63	41	55	37	63	71
Began training at age…	7	9	25	13	14	8	11	20	12	7	16	8	32
Began curing at age…	29	15	30	58	22	15	25	25	30	32	25	13	34
Overall years of clinical experience	44	31	24	14	14	35	28	38	11	23	12	50	37

### Key epistemic concepts

**Fig 1:**
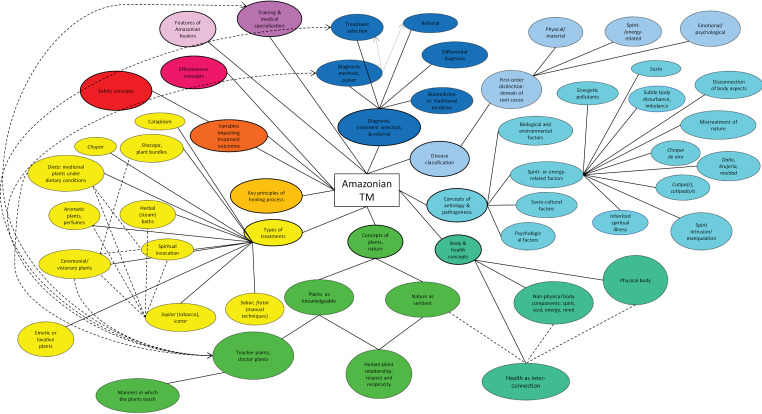
Mapping epistemic concepts in Amazonian traditional medicine (TM).

Fig 1 provides a conceptual map of themes and sub-themes that emerged in the content analysis, yielding a total of 12 main clusters that describe key epistemic concepts in contemporary Peruvian Amazonian healing. Since a detailed description of the full list would be beyond the present scope, the current work will focus on seven clusters: (1) *Body and health concepts;* (2) *Concepts of plants in the healing context;* (3) *Disease classification;* (4) *Concepts of aetiology and pathogenesis;* (5) *Diagnosis, treatment selection, and referral;* (6) *Overview of types of treatments;* and (7) *Effectiveness concepts*. The remaining emerging clusters (*Patient safety, Key principles of healing process; Variables impacting treatment outcomes; Features of Amazonian healers; Training in Amazonian medicine and medical specialization*) will be reported in a separate paper. In the accounts that follow, we will describe in particular detail aspects that can be subsumed as spirit- or energy-related, given that they represent concepts entirely absent from Western healthcare epistemologies, yet, as per our informants, are especially fundamental for understanding the Amazonian view on illness and treatment.

#### 1. Body and health concepts.

The descriptions of the informants suggested the human body to be composed of a number of structural elements, which in our analysis could be broadly divided into physical/material and subtle/nonmaterial body components. Health was often articulated in terms of correct alignment and association:

***1.1. Physical/material and subtle/nonmaterial body components:*** Subtle or non-material components of the body referenced by the healers included the spirit body, energy body, soul, and mind (some healers referring to only a subset of these terms), for example: “Us healers we know the spirit, we know the soul, and we know the physical body […] the soul and the spirit are the parts that are not visible to the human eye. That is, ordinarily they are invisible, but if a person has specific training, they can see. Thanks to the plants it is possible to see at the level of energy.” *HF*. Specific locations on the physical body were described as interface or access points to subtle body structures, for instance as *HG* mentioned, entry points located “at the crown of the head, on the hands, chest, upper back.” The healers described nonmaterial body aspects as more fundamental than the physical: “The spirit body is like the support of the physical body; if your spirit-body is weak, your physical part will not function properly. If it is weakened, for instance when the subtle body receives a strong charge and cannot properly sustain or carry the physical body, then physical symptoms can appear, like pain, weakness; or negative feelings, sadness, anger, fear.” *HB*. Illness of subtle body components may thus manifest as physical or emotional symptoms.

***1.2. Health as interconnection:*** Overall, good health was explained to depend on the intactness and correct alignment of the above components (“If the soul is correctly placed in its location in the body, the person is fine” *HE*), and Amazonian treatments intervene to align, heal, and cleanse these, e.g., “Natural medicines like tobacco and other plants clear the energy channels, the channels open and the energy can flow upward and downward.” *HK*. During treatments patients may sense this “as vibrations in different parts of their body, like an electric current” *HB*. Good health was further described as based on interconnections beyond the person’s own body, relating to aspects of the natural and social environment: “The spirit, soul, and physical body, the three connected, also have a connection with the elements […] Without the elements the human being dies –if the body has no water it dies. Without appropriate heat, fire, it also dies, and likewise with no air. So the human body connects to the elements for its sustenance; via the connections with the elements, you are thus connected to nature.” *HF*. Another example was: “Seeing the mountains, the peaks, the rivers, I feel something pleasant – this means to be connected. […] All of a sudden you may see the stars, planets, the entire universe as something marvellous and feel that you are part of it. The fact that you sense the stars, the sun, moon, everything as part of yourself, this sensation of joy, of happiness - it is healing.” *HC*.

#### 2. Concepts of plants in the healing context.

In our analysis the informants invariably referred to plants as sentient, knowledgeable, and capable of teaching humans, also emphasizing the kind of relationship that is important in this context:

***2.1. Nature as sentient:*** The healer accounts described nature as sentient and conscious: “we conceive it that way because we can directly sense that all beings have a spirit. The spirit of some beings of nature may be smaller, more subtle, less powerful or more delicate, one could say, while other spirits are more potent, stronger. This is very different from saying ‘only us human beings are there and all the rest are mindless things’.” *HC*. Certain natural localities and plant species are further understood to have guardian spirits, also referred to as ‘owners’ or ‘mothers’ (e.g., “There are plants where you can see small spirits below, protecting its trunk.” *HI*; “the rivers have a mother” *HD*; “the *Yashingo* [name of the forests’ guardian spirit, also called Chullachaqui, Shapshico, etc.; see [113,114]] is the owner, he protects everything in the forest, the animals, plants.” *HA*). The existence of such guardian spirits frames the way the healers relate to nature (see *2.3.*). They explained that the spirits dwell particularly in remote parts of the forest: “Outside of the cities, deeper in the forest, the spiritual aspect is more open and one encounters a lot of spirits.” *HB*. The impact of population growth and increased migration into Indigenous territories was also addressed in their descriptions: “In the past I used to walk three hours into the forest towards the ridge, and there were strong energies. Now as the population has expanded and reaches into this region, the spirits have retreated deeper into the forest, they have moved away.” *HG*.

***2.2. Teacher plants, doctor plants:*** As with nature overall, plants were described as conscious (“Each plant is like a person, they are different from each other” *HB*) and often as knowledgeable, or wise: “the trees are our older siblings, so as our older siblings they come to our help, to guide us and help us in accordance with their specific capacities” *HF*. Some plants were commonly referred to as ‘doctors’ (“Each plant is a doctor”, *HB*), as ‘teachers’, or ‘masters’, due to their described capacity to instruct humans in various types of domains and skills: “They cure you, they heal you, they teach you–they are teachers. I have a lot of trust in them, I feel very grateful to them” *HA*. The latter quote also alludes to the kind of relationship healers develop with the plants; a feeling of gratitude towards somebody that has helped/can help you. While some healers defined the teacher plants in terms of specific qualities (e.g., “these are the plants or trees that have a very strong healing force and have the capacity to teach you medicine” *HE*), others employed a broader perspective (“In my view all medicinal plants are potential teachers, not just one; all plants may teach you.” *HM*).

In the healing context, the plants may teach a healer the practice of medicine (e.g., impart knowledge about medicinal plants and their applications, for instance: “Each plant has a spirit, and, in accordance with the *dieta* that you are following, they reveal things to you. They may tell you ‘take this plant or do such and such thing, and you will get healed’–this is the spirit of the plant telling you” *HH*). Further, the teacher plants may teach about the subtle body components or the energy and spirit-aspects of nature described before: “Here in the Amazon we ingest the plant, eat it, drink it, and thereby enter into contact [with it]” *HC*. Further, “The teacher plants are the ones that can be dieted in order to know the realm of energy” *HB*. Ingesting these plants under specific dietary prescriptions, “they connect or open or awaken you to the spirit realm. The human body will suffer certain discomfort in this process, depending on the preparation; they help the spirit body to awaken and walk” *HF*. As some of the quotes reveal, a set of nutritional and behavioural prescriptions locally referred to as *dieta* are considered indispensable in this context (see section 6. for teacher plants and the *dieta* as therapeutic tool).

Further, the teacher plants may teach about different life domains, for instance offer patients insight regarding antecedents of their illness, relationships, etc. The informants mentioned various ways in which the teachings from the plants are conveyed, including dreams (“You take the plant remedy and in the dream the plant will explain things to you” *HI*), visions (“I see it like a movie in my mind, it used to happen quite regularly; what had occurred, what should be done, like a movie.” *HH*), revelations (“the medicinal plants give us revelations, they give us visions, the spirit talks” *HJ*). Or as *HC* reiterates: “These plants are called teachers because they really teach; the way in which they teach varies, it can be through sensations in the body, through spontaneous insight, or the plant comes and tells you in your mind ‘you should do this’.”

The practice of Amazonian medicine is thus rooted in a healer’s relationship with specific plants. The healer–plant relationship is established through an extensive and often strict training process: “after you do the traditional training, [which involves following] many diets, the spirits of the plants are with you.” *HE.* While each healer works with a personal set of plants, their work tends to be informed by a main teacher, which defines their medical specialization: for example a healer whose main teacher is the tobacco plant (especially *N. rustica*) is designated as *tabaquero/a*, one whose main teacher is ayahuasca (*B. caapi*) is referred to as *ayahuasquero/a*, or a healer who relies mainly on trees (*palos*) is called a *palero/a*, etc. “The teacher plants are the ones that teach you the medicinal plants, how to cure; no other persons have taught me, my father has initially guided me and given me knowledge, but who has given me most knowledge were the plants” *HI*.

***2.3. Human-plant relationship: Respect and reciprocity:*** Starting with the process of harvesting or collecting the plant, through to treatment delivery, an attitude of respect and reciprocity is stressed in Amazonian medicine. Asking the plant for permission to take from its bark, leaves, or resin, and offering something in return, are considered essential. *HD* for instance stressed that before collecting the plant “the healer will speak to the plant, they will ask, offer a prayer, and the plant will understand; because just like that you cannot take it”, which was also expressed by *HJ*: “one has to offer a payment, you don’t just take”. Similarly, for the process of preparing and administering the remedies, the healers highlighted that the act is not merely utilitarian, but rather based on reciprocity: “You are in a relationship with the plants, you ask them to heal the patient” *HE*; or as *HJ* explained: “one has to ask for them to give, to heal”. *HA* mentioned the importance of gratitude, in acknowledgement of the support received: “In my ceremonies I give thanks to the plants, ‘thank you *maestro* for being here […] please take care of this patient’.” *HA*. In this sense, adhering to the dietary prescriptions is also understood as a sign of respect, as “a commitment to the plant” *HE*. Breaking the commitment has consequences: “the plant protects you and unprotects you. A person drinking the remedy should give importance to the diet requirements. When the plant is with you it protects you, but if you exit the frame of the diet rules, the plant lets go of you and harm can come to you.” *HI*.

#### 3. Concepts of aetiology and pathogenesis.

While the descriptions of the healers in general suggested origin and development of diseases as complex and multi-causal phenomena, they offered a range of examples for causal contributors, which in our analysis could be grouped into three broad domains:

***3.1. Environmental and biological factors:*** Newly adopted alimentary and agricultural practices in the region, as well as waste products from mining activities, were identified as contaminating foods and harming the body. Nutrition in general was stressed as a key factor for health and the development of illnesses: “One of the causes is our nutrition, our food. You should listen to your body […] and we need to eat natural products; the chemicals are a problem” *HM*. Some healers used the expression ‘e*stomago sucio’* or ‘*intestino sucio*’ to refer to an accumulation of waste or toxic material in the intestines or stomach, which was pointed out as a fundamental causal factor that may lead to different types of illnesses, including mental health problems: “when this [pointing to the belly] is contaminated, your mind goes awry as well, you don’t think clearly” *HA*. The informants further mentioned parasites, viruses, or bacteria to cause illnesses, as well as lack of hygiene. Furthermore, accidents were mentioned, as well as carrying heavy loads for people working in the *chacra* (small farming plots in the forest), which makes them prone to develop *lisiados* (literally: bent, hurt, in pain), a common musculoskeletal condition. For urban dwellers it may also ensue “due to the bad posture of sitting a lot of time at a desk” *HL*. Finally, prolonged exposure to cold water or wind was also mentioned as a contributor to illness, for instance regularly washing clothes by immersing one’s hands in cold water.

***3.2. Socio-cultural and psychological factors:*** The informants further mentioned social circumstances and psychological factors to impact health. For example *HE* explained: “I think one of the causes are the pressures on people from their culture, and from what happens in politics […] or drawing from their own past”, or: “for some people it is due to stress, related to how they live” *HB.* Violence and traumatic events were pointed out to be associated with ill health (e.g., “different types of abuse, the person’s mind is left tormented” *HB*), but also personal psychological tendencies, such as excessive worry and rumination: “thinking, thinking, thinking how to solve a problem, it causes fatigue in mind and body” *HE*. One healer explained this as follows: “We tend to listen to our mind instead of our body, something we lack proper coordination of. We usually think and attend to the mind’s thoughts, ‘I want this, right now’, instead of attending to what the body really needs. […] Thinking is good, a lot of thinking is not useful; not thinking at all is also no good – we have to look for a balance in thought.” *HM.*

***3.3. Spirit and energy-related factors:*** The healers described factors that can be subsumed as spirit- or energy-related influences as particularly essential in pathogenesis. For example, energetic pollutants may accumulate in the subtle body: “over time, the energy field of a person may become like a trash dump, and in a trash dump you find all sorts of things. […] Sources of contamination there are many; for example technology, cell phones, televisions, even cars – they are very useful, but for the energy body it is toxic. Another common type of energetic pollutants comes directly from humans, from their mentalities; bad words spoken about others. In this way energetic waste accumulates in the body. If it is not cleaned in due time with the help of the natural medicines, this load results in illness.” *HF*. The description also alludes to the concept of cleansing of the energy body, which is stressed in Amazonian healing for both prevention and treatment of illnesses (see also section 6. on emetic plants). Other types of disturbances include imbalances in the subtle body, for instance “mental imbalance that arises due to an excess of energy [in the head], or an oversensitivity arising from a too open energy” *HM*. Furthermore, a sudden shock referred to as *susto* may displace a person’s vital energy from its locus in the body, which can lead to symptoms (diarrhoea, insomnia, anxiety). Children were described as especially vulnerable to *susto*, but if promptly treated with the appropriate traditional techniques and plants, the condition can be remedied easily. However, if untreated, childhood *susto* is described to act as a causal factor for adult psychological problems: “In the case of foreigners, possibly 100 or 80 percent of them may have *susto*. Because in their cultures these things are not known [and therefore not treated], so the child grows up, becomes a man or a woman, but the *susto* is evident in their behavior; a lot of anxiety, lack of self-confidence. This is because they grew up without being cured; it doesn’t kill the child, but energetically it is very harmful and manifests in adulthood. Here in the Amazon it is different, people don’t exhibit this kind of anxiety because from early childhood on they were cured in case of *susto*.” *HF*; “It is the echo of the *susto*, from there [later in life] emotional problems can arise. It begins with something, an accident, abuse, anything. If untreated, emotional problems begin to form, like a time bomb” *HM*. Also more generally, a disconnection between physical and subtle body aspects is considered to cause health problems: “Problems and illnesses arise when these aspects are divorced, separate, ill-accommodated. For instance the way a person thinks is separate from their corporeal reality” *HC*. “When you are disconnected, the spirit here, the body there, this is associated with illness; you may feel a lot of anxiety, or you may sense an inner void, there can be different symptoms […]; the cause is that body, mind, and spirit are not properly connected.” *HM*.

In other cases, illness may be caused by foreign spirits. An example is *choque de aire* (also: *mal de aire)*, which refers to an impact by a bad spirit (“mal aire is when you perceive a bad spirit” *HL*), which can lead to different symptoms, such as a “decompensation of their body, their energy … the person may get pale, first they have shivers, then nausea, vomiting, diarrhoea, strong fever.” *HB. HK* recounted a recent case of an elderly woman: “The grandson came to get me, I went to see her, she was like this, her neck was twisted and she was unresponsive. The family members called her name, ‘Janet, Janet’, but she didn’t respond. That can happen due to a *choque de aire.*”. A related syndrome is *cutipa(r)* (or as adjective: *cutipado/a*), which involves an encounter with a foreign spirit, one that has a strong force that may impact the human. For instance specific animals (e.g., sloths, certain monkeys, river dolphins, *sachavacas* [Amazonian tapirs]) may *cutipar* the person, as a result of which they may develop symptoms like diarrhoea or vomiting, but also exhibit some feature of the animal in question energetically transposed to the person (e.g., a child *cutipado/a* by a sloth may develop a sleepy face or slow movement, in a pregnant woman it might slow the birthing process). As with the former, children and infants are considered most vulnerable, including prenatally via the pregnant mother or in some cases via the father through consubstantiality. Another type of *cutipa* that leads to transposition of features was described to result from eating certain foods that are to be omitted during pregnancy or during a plant-diet (e.g., swelling of the belly after eating pork meat). Further, mistreatment of nature can also lead to illness, for example overharvesting or cutting down trees: “There are plants that do not agree to be mistreated; the plant may respond by harming the person, the person may become gravely ill” *HJ*. Furthermore, the informants described cases of incorporated spirits associated with manipulation: “there is a type of being that can enter the body and manipulate the person” *HC*; in such cases “you don’t really do what you want, but the entity is manipulating you; the person becomes like a marionette” *HM*.

Finally, the healers mentioned *daño* (also: *maldad, brujería*, *mal de gente*, or *hechicería*), referring to deliberately inflicted illness through sorcery, as a possible causal factor: “Practically it is an illness that another person is doing to you” *HL*; “*Brujería* [sorcery], someone pays a *brujo* [sorcerer] to make their enemy fall ill.” *HF*. “Someone that knows how to do *maldad* [sorcery] will use your energy” *HG*, or “they use the plants, for example in *pusanga* [love spells].” *HJ*. “The ones that do this here in the Amazon, we call them *brujos*, they utilize the spirits to cause harm to others. A common example would be, let’s say a couple with children, the husband has a job, a salary, but he is a womanizer and has another woman. When the wife finds out she confronts him, and he decides to leave and go with the other woman. So the wife will be angry and will want some sort of revenge. […] She goes to a kind of shaman that is not good, he’s not a *curandero*, but a *brujo*, and she will tell him her story and say ‘I want you to get this woman out of the picture’. Since he’s a *brujo* and wants money, he will accept. He will utilize the spirits or energetic tools to cause harm to that woman” *HE*. *HC* noted that “a *brujo* that deliberately causes harm may consider himself powerful, but, in reality, he is just a marionette of another, more powerful instance”, alluding to the aforementioned manipulation by negative spirits. Finally, as some healers mentioned “a person may also inherit from their parent a spiritual disorder, an ancestral *daño*” *HC*, akin to the inheritance of a physical or mental illness.

#### 4. Concepts of disease classification.

While the previous section touched upon multifactorial causes of disease which may serve as basis for classification in itself, the healers also often used a broad distinction, which in our analysis involved three general clusters:

The informants distinguished conditions that are physiologically rooted (e.g., hernia, urinary tract infection), conditions rooted in the spiritual/energetic domain (e.g., *daño*, *cutipa*), and conditions rooted in the emotional/psychological domain (e.g., relationship problems, stress). These broad disease categories were often used in the discourse as a first-order classification of diseases. However, although important for treatment selection, the distinction is not as clear-cut as it might suggest, as a given health condition may in principle include a combination of physiological, spiritual, and psychological aspects at once. As an example, a bodily symptom like diarrhoea and vomiting could potentially stem from an underlying physiological illness (e.g., food poisoning), but it could equally derive from an underlying spiritual/energetic illness (e.g., *choque de aire*), which would require a fundamentally different treatment strategy. This complexity may be usefully contextualized by the previously described healer accounts regarding co-existing physical and non-physical structural elements of the human body, which interact with each other and the external environment in health and pathogenesis.

#### 5. Diagnosis, treatment selection, and referral.

The healers provided detailed descriptions of the various stages of their service leading up to the treatment, including diagnostic methods, decision-making processes regarding treatment selection and referral, as well as the complexities of collaboration between traditional and biomedical healthcare:

***5.1. Diagnostic methods:*** The informants described the diagnostic assessment to begin with a conversation: “I ask them about what happened to them, for how long; I ask them questions in this way, and simultaneously I mentally examine their body” *HL*. Some informants mention examining the eyes, tongue, or body posture, and particular attention to the quality and flow of the patient’s energy. A common traditional diagnostic method involves the palpation of the pulse: “We touch them here at the pulse, and watch, and sense. Since we have been trained by the plants we can sense what the patient has in that way, what they need; *pulsar* we call it” *HI*. Similarly, *HF* explained: “With the help of the pulse and my mental eyes I examine their entire body and see which illness they have, in this way I know how to treat them. The pulse is like a vehicle to see the body”. In addition, a healer’s allied teacher plants may inform the diagnostic process, for example: “A healer taking the pulse observes with the help of the tobacco, this is what happens. By smoking the tobacco the healer can see the person.” *HJ*; or: “I make a first diagnosis based on my observations, then to remove any doubts we drink ayahuasca, the plant tells me what the problem is.” *HG*. This may involve trance states in which diagnosis and treatment indications are revealed to the healer. The example of toé was given by *HJ*: “Within ten minutes it gives a strong *mareación* [dizziness/altered state] in which you may discover thefts, or which illness someone has; doctors may appear who tell you ‘that man or that woman has such and such disease, and it can be cured with this’; based on this the healer treats the patient.”.

***5.2. Differential diagnosis in uncertain cases:*** The capacity to distinguish between disease categories is seen as a critical skill of a traditional healer, acquired during years of training and direct clinical experience. Cases of diagnostic uncertainties can however occur, for which some healers report consulting their teacher plants, for instance “if there is a person with a problem that the healer cannot clearly identify […] I use tobacco, the pulse, and as a last resort ayahuasca.” *HE*. For differential diagnosis between inflicted and natural illness the following procedure is often described: “when the person has *hechicería*, we can detect it at their hands or feet. It is a sensation like rays, or electricity, it rises up through the hand if we touch them.” *HB.* Similarly in *HL*’s example: “When the body is clean [from *daño*], you touch it and nothing happens. But if I sense *tssss*, a shiver that enters my hand, this is not for a biomedical doctor, not for a huesero – this is for a *curioso* [local term for healers that train or work with teacher plants]”. *HF* reports encountering also the opposite situation: “Some people come to see me convinced that they have *brujería* [witchcraft], but then I check them and they have no *brujería* whatsoever, it’s a physical problem”.

***5.3. Treatment selection:*** After the diagnostic assessment, the treatment is typically tailored to the patient at hand. In the decision process for selecting which treatment approach to use, a healer may take into account the patient’s level of severity of illness, his or her economic situation (“for example in case of a broken bone I usually send them to the doctor for a cast or surgery, but if I see that they cannot afford this financially, I will treat them, asking them only to buy the ingredients.” *HH*), as well as the overall condition of the patient and possible contraindications for certain treatment methods (e.g., “if the person is too sick on a physical level, they may not be able to sustain an emetic plant for instance; we start with plant baths” *HE*).

***5.4. Referral:*** Like in the above example, informants described cases in which they would refer patients to other types of health professionals, for example “if the body has other types of problems that are better addressed with chemical medicines, you send them to the clinic” *HA*. One informant explains referring patients to a biomedical doctor “if spiritually and energetically there is nothing wrong with the person, but physically they are in a very bad state; in such cases a doctor may be able to help promptly” *HF*. Or: “this is why we sense the pulse, if we cannot treat them, we send them to a hospital” *HI*. In some cases, it may be the teacher plant that suggests a referral: “If the plant reveals us the hospital, the patient needs to be treated there” *HJ*. The informants further recounted cases in which they would refer patients to other traditional practitioners, depending on the healer’s area of specialization: “If the problem is just in the body, I can cure it, but if it is *daño* I prefer sending them to a *vaporadora*; I tell them ‘I am afraid to treat *daño*, go to a *vaporadora*’.” *HD*.

***5.5. Biomedicine vs. traditional medicine:*** As evident in the prior descriptions, healers regarded traditional and biomedical approaches as complementary to each other, generally considering each system to have its benefits: “for instance strong ear pain; we can heal this with plants, but it may take three days, while there are pharmaceutical medicines that can alleviate the pain within half an hour. Although the problem may not get cured like that, it just gets anesthetized, but for strong pain this is useful” *HF*. For energetic or spirit-related health conditions on the other hand, the healers considered the biomedical paradigm uninformed (“An example, a person out of the blue gets seriously ill, the doctor examines them with all sorts of analyses but finds nothing, the physical body is healthy. But it is evident that the person is in a critical state.” *HF*). Pharmaceutical treatments are said to be ineffective and may even aggravate the condition (e.g., “if a patient suffering from strong *brujería* is given pharmaceutical medications, their state quickly deteriorates, they die.” *HF*). Furthermore, the healers mentioned that while pharmaceutical treatments often focus on alleviating symptoms, “the plants go deeper, they cure you. The plants have the added benefit of working in the spiritual dimension. They help you to listen to your body, your emotions, your sensations.” *HE.*

For physical conditions, instances of indirect collaboration were sometimes described, for example one healer recounted cases of patients that had beforehand been diagnosed with tuberculosis or HIV, for whom they prescribed specific herbal remedies within traditional *dieta* frame, but asked them to assess treatment progress with periodical laboratory tests.

Despite the complementarities, the participants explained that traditional healers are at a disadvantage, since their contribution is not acknowledged by the official health system: “Clearly only the medicine system of the doctors is recognized, they work within the recognized medicine and the laws protect them. For instance if a patient dies in their hands, there are laws that protect them. This is not the case for a *curandero.*” *HF*.

#### 6. Overview of types of treatments.

While the range of treatment types a given healer uses in their clinical practice depends on their medical specialization and other idiosyncratic factors, they often pointed out the diversity of Amazonian plants and described sophisticated application methods. In our analysis, the main therapeutic methods used by the healers included the following:

The aforementioned *dieta* or diet method, aside from its importance for a healer’s training, featured prominently in the informants discourse, who referred to it as a fundamental therapeutic tool in Amazonian medicine (“for me, any kind of healing requires the *dieta*, there is no other way” *HE*). The *dieta* frame in general is said to maximize therapeutic benefit and provide essential safety for the ingestion of teacher plants, while the specific dietary protocol (duration, restrictions, dietary regime; e.g., “we give them food without salt, without oil/fats, without spices, and without sugar.” *HM*) varies depending on the illness at hand or herbal remedy used. Another characteristic Amazonian treatment modality involved traditional (ritual) application of emetic/laxative plants, commonly used for physical, energetic, and emotional depuration (*limpiar*) to restore or maintain health. Further, some healers reported working with visionary plants in ceremonial contexts, employed for both diagnostic and treatment purposes. As with the *dieta* frame, ceremonially applied plants were described to have therapeutic effects on multiple levels, affecting physiological, spirit- or energy-related, and psychological parameters. For example, the visionary plant “offers a doorway to the spirit realm, where the person can connect with spirits, they may see things about their own life, the past and the present, how you live life; and also about the future” *HK*; or “the plant herself heals you; it alleviates emotional problems but also cures witchcraft.” *HH*. Topical application of plants via herbal steam baths or showers were another common method, with different plants used depending on the ailment to be treated. In some cases also cataplasms were described. Furthermore, the tobacco plant was referred to as essential for the process of curing, be it as an offering, as a liquid medicinal preparation for ingestion, or blown in smoke form (*soplar*) on specific points of the patient’s body. “Tobacco acts like a repellent for negative spirits.” *HI*. *Soplar* is considered a critical tool, but “for the blowing that is externally observable to actually have an internal [therapeutic] effect in the patient, the healer transports their healing force; like this the negative gets cleared from the body, and the body of the person becomes balanced” *HF*. Similarly, a technique referred to as *icarar* is considered key. *Icaros* are “special chants that move energetic fields. There are different *icaros* with different purposes” *HF*; “with the *icaro* we call the plants, so that the spirits of these plants come and purify or heal the patients” *HJ*. Furthermore, healers may perform ritual cleansings using bundles of fresh or dry herbs (e.g., *shacapa*) along with the *icaros;* some informants also reported using instruments like the *maraca* in ceremonies. Specific aromatic plant extracts or perfumes were described as part of healing rituals. Spiritual invocation or prayer was further mentioned as inherent in a healer’s work, although not necessarily discernible for external observers. Furthermore, a traditional method for extracting energetic pathogens from a patient’s body via suction (*chupar*) was also reported by some healers. Finally, manual techniques on a patient’s body *(sobar, frotar)* were described either in conjunction with one of the former treatments, or in case of bonesetters as main treatment modality.

#### 7. Effectiveness concepts.

To know if a treatment has been successful the informants mentioned monitoring the process: “A *curandero* has to be in continuous communication with the patient throughout the treatment. If they feel something, if something hurts, or does not hurt anymore; do they feel good or bad; because people may respond to treatments differently, and the curandero has to adapt the methods accordingly.” *HF*. “If a patient tells us ‘I’m hungry’, when before the treatment they had no appetite, this means that they are getting better.” *HJ*. One informant explained that it “is easy to know [if the treatment has been effective], you observe it on the patient; the person displays a calmness and a joy, which the healer then also senses. So these are the indicators, joyfulness and tranquillity. They have more trust, the pain goes down, they feel happy, and this joy is felt also by the healer - it is quite beautiful.” *HH*. Or in another healer’s words: “the face looks different, the attitude is changed; it is quite obvious” *HC*. Another informant explained that “In the same way as when we started the process, also at the end of it we have the capacity to sense how the patient is.” *HB*. Similarly, *HE* considered they also may know how the patient is progressing “based on our connection with the spirits”. For some physiological conditions it was considered sensible to confirm treatment success with laboratory tests: “I tell them ‘once you have finished this remedy, go to the health center to take the analysis’.” *HD*.

## Discussion

Ethnically-based inequalities in healthcare are a persistent problem in Latin America, including Peru as one of the countries with the largest Indigenous population. Health disparities are particularly striking in the Peruvian Amazon region [29–36,55,115] making up 60% of Peruvian territory (and 40% of South America), and adjacent countries with related Indigenous population (e.g., Brazil, Ecuador) show similar inequalities and lack of culturally appropriate healthcare [44,116,117]. The first formal effort by the Peruvian Ministry of Health to recognize the importance and need to respect Indigenous medical traditions through a policy document stems from 2005, which was however limited to a very specific context (maternal position during labor and childbirth) [118]. Since then, ten other governmental documents to enable the use of intercultural health approaches at the primary health care level have been issued (for a list see Table 1 in [47]). However, these regulations so far did not translate into care models in which Indigenous medical perspectives are at the core. Instead, practices were cherry-picked (e.g., position during child delivery), and the necessary actions to implement regulations, including the documentation of Indigenous medical perspectives, or the provision of training on cultural sensitivity for healthcare workers assigned to Indigenous districts, are still missing [47]. Aiming to support Indigenous Organizations and governmental initiatives that promote inclusive healthcare and Indigenous peoples’ right to have their perspectives represented in public health services, which in turn contributes to UHC [19,21,23,39,46,53,54,119], the current study investigated key epistemic concepts in traditional Amazonian medicine based on in-depth interviews with accomplished traditional healers from three regions of the Peruvian Amazon.

Our findings point to an intricate and complex traditional knowledge system that involves multifactorial concepts of illness and aetiology, health, diagnosis, and treatment. The latter include sophisticated medicinal plant applications in conjunction with carefully designed traditional techniques. While the relevance of pharmacological mechanism in conjunction with medicinal plants is clear, the healers also emphasized a spirit-aspect of plants, based on their understanding as sentient. In this view, plants are not conceived of as objects, but as ‘other-than-human’ persons (see also [120,121] for this concept), with whom relationship and communication can be established. The informants pointed out that this understanding of plants was derived from their direct experience rather than from belief, describing specially designed traditional techniques that render the spirit-aspect of plants accessible to human perception and interaction. Some plants were described as teachers, who, under the conditions of respectful and reciprocal relationship, may instruct humans in various forms and domains. Indeed, the concept of plants that teach is at the heart of this medicine system (see also [105,122]), informing the healers’ clinical practice from training through diagnosis and treatment (Fig 1), as well as patients’ insights during their treatment process. In this context, the *dieta* technique is a distinctive feature of Peruvian-Amazonian medicine and a unique method for the application of psychoactive and other medicinal herbs [107,123–125]. In addition, the ecological merit of the Amazonian view regarding plants and nature is evident, given the implications for behaviour and its acknowledgment that human health is intimately linked with the health of the natural environment (see also [113,114,126]). Interestingly, the healers pointed out that with the growth and expansion of population into forest territory, the local spirit landscape has become impoverished, a phenomenon that might go hand in hand with spreading deforestation, threat to ecosystems, and species diversity [127–130].

Further, epistemic concepts relating to bodily aspects not visible to the untrained eye are said to play a fundamental role in disease aetiology as well as in therapeutic intervention. However, far from offering simplistic accounts based on magical causalities as sometimes suggested in early anthropological writings (see e.g. [131–133], the healers describe complex interactions between material and nonmaterial causal factors with observable effects on health. The informants point out refined techniques (e.g., *pulsar*) to distinguish between types of illness, the mastery of which requires a long-term training process. They mention therapeutic interventions that target specific physical, emotional, or spiritual/energetic conditions, but also plant-based ceremonial techniques described to act at the juncture of body, mind, and the subtle, affecting multiple domains simultaneously. Indeed, the nexus between body and mind is not yet well understood in contemporary health sciences [134–136], and explanatory models from Indigenous knowledge systems may well be worth taking a closer look at. The concept for instance of *intestino sucio* (filthy intestines) as a bodily condition that impacts psychological parameters conforms with cutting-edge scientific evidence of the relationship between gut microbiome and mental health (gut-brain axis) [137–139]. The emphasis on emetic/laxative plants and diet in Amazonian medicine [106,140,141], and perhaps also in other traditional healing systems [142–144], could indeed be targeting this axis. Further, the concept of *susto* was mentioned by the healers not only as a health conditions in itself, but also as a cross-culturally relevant risk factor for adult psychopathology. *Susto* is a common illness concept across Latin America, albeit under different names [145,146], and is also described in the current version of the *Diagnostic and Statistical Manual of Mental Disorders* (DSM 5) [147], where it is listed under ‘cultural concepts of distress’. The manual however also states that “similar etiological concepts and symptom configurations are found globally” (p. 836) [147], thereby, much in line with our informants’ accounts, suggesting cross-cultural occurrence.

In addition, our findings point to profound knowledge regarding psychoactives and their clinical applications in Amazonian medicine. The healers’ descriptions of how they understand and work with psychoactive herbs and associated altered states of consciousness reflects unique experience, orally transmitted over generations, and reaffirms the importance of traditional healers’ inclusion to clinical studies of the current psychedelic revival, in view of both leading expertise and intellectual ownership [76,77,148,149]. Their advice and participation in newly emerging training programs for ‘psychedelic therapists’ [e.g., 150,151] may be particularly important. Our informants underlined the necessity of specialized and long-term training for safe administration of plants like ayahuasca, in the form of extensive traditional retreats during which the aspirant’s body, mind, and spirit get prepared for the risks and challenges of the task, and relationships with the Master Plants are established. Short-cuts in training were described as hazardous for both patients and therapist (see also [123,124,152,153]). Interestingly, although the currently most famously discussed plant remedy in this context is ayahuasca, the healers also prominently emphasized the tobacco plant as Master Plant and fundamental tool in their work, describing applications in stark contrast with recreational smoking. Indeed, among Indigenous groups across the Americas tobacco is known as a sacred herb [154–158], and it was only after the colonizers brought the plant to Europe (late 15^th^ century) that today’s commercial tobacco products with the known deleterious health consequences were developed and globally marketed [158,159]. In Amazonian medicine, however, knowledge regarding medicinal applications of this plant have persisted, the centrality of which can be considered another distinguishing feature of the Peruvian Amazonian healing system. Indeed, in several Indigenous languages of the region (e.g., Matsigenka, Yuracaré) the generic term for ‘healer’ is etymologically linked to the word for ‘tobacco’ [63,160–163]. Clinical research on Amazonian therapeutic uses of tobacco has only begun to emerge, but suggests promising first findings in the mental health domain that warrant further investigation [89,141,164].

Our findings show traditional healers recognize complementary advantages of both traditional medicine and biomedicine, yet, in line with other reports [39,165], identify the lack of recognition and respect for their profession and expertise as a barrier for collaboration. Indeed, preconceptions and stigma on Indigenous medicine, along with a paucity of research, still represent an impediment to countries’ ability to respond to Indigenous peoples’ health-related expectations and needs, thus maintaining existing inequalities. Long after the institutionalized persecution of Indigenous healers has ceased [166], the region’s colonial legacy continues to impact societies across Latin America, perpetuating discrimination and exclusion of Indigenous medicine actors and complicating the efforts of Indigenous Organizations and local governments to establish equitable healthcare for Indigenous populations towards UHC [13,32,167]. Clearly, calls for legitimization of Indigenous medicine, such as the current, run against deep-rooted historical processes that will take time and effort to shift. As one of these, the epistemic concepts mapped in this study can be used to articulate collaborative models that bridge the gap between traditional healers and biomedical actors in routine care services, and, within this and similar contexts in Latin America, be instrumental for the efforts towards inclusive healthcare.

The current study has several limitations. First, a map is not the territory, and to some extent inevitably simplifies a complex and vast terrain, which merits further in-depth study. Second, while we attempted to provide some key concepts of Amazonian medicine, the reality of traditional healing practice includes idiosyncrasies between individual healers and ethnic groups. However, some characteristic practices and underlying principles are common between healers (e.g., concept of plants as sentient, *dieta*, *icarar*, *soplar*, etc.), and it is those shared concepts the current work focused on, aiming to provide a broad and cross-ethnically relevant map that can support dialogue between medicine systems. Finally, while our sample size was well within the norm of qualitative and ethnographic research [84,168], it included informants from only a subset of local Indigenous groups. Nonetheless, considering the most prevalent linguistic families of the region as a proxy (Arawak, Jibaro, Pano, and Quechua) [169–173], the main Peruvian Amazonian cultural clusters were well represented in the sample.

## Conclusion

The current work highlights profound Indigenous knowledge linked to medicinal plants, integral health and illness concepts, and a sophisticated, multilayered treatment approach practiced by traditional healers of the Peruvian Amazon. It also highlights Amazonian healers’ unique expertise in the therapeutic application of psychoactives, from which academic health sciences may have a great deal to learn. As such, the work calls for the inclusion of Indigenous medical perspectives (including the epistemic concepts presented in this work) and traditional healers to public healthcare within this and similar regions, as well as for their inclusion to scientific research and emerging clinical paradigms in conjunction with the psychedelic renaissance.
